# Supplementary material for: Home range size and habitat selection of owned outdoor domestic cats (Felis catus) in urban southwestern Ontario
Source: PeerJ. 2024 Mar 29;12:e17159. doi: 10.7717/peerj.17159 (PMC10984174; doi:10.7717/peerj.17159)
Supplement: Supplemental Information 1 [file peerj-12-17159-s001.pdf]

Many owners put their cats outside for a variety of reasons, such as alleviating boredom, getting exercise, and allowing their cat to engage in a more natural behaviour. Unfortunately, leaving a cat outside and unsupervised exposes them to many risks. Some of these risks include acquiring and transmitting diseases, being attacked by other animals, and being hit by a vehicle. But what many cat owners don't know is that every year, unsupervised outdoor domestic cats (*Felis catus*) are believed to kill billions of wild animals worldwide. Despite this, there is uncertainty in the scientific literature as to what species of wild animals are hunted by domestic cats and how many animals are captured throughout the year.

In this research, we will use a small, lightweight camera that attaches to a cat's neck collar (the '*Catcam*') to determine the number of birds, amphibians, reptiles, and small mammals captured by owned cats that are allowed unsupervised outdoor access in the Kitchener-Waterloo-Cambridge-Guelph area during different parts of the year. Our research will give us a better understanding of what cats do outside when nobody is looking and can help cat owners make better-informed decisions about their cat's health.

These questions will determine if you and your cat are eligible for this research.

You may withdraw from the survey at any time.

This project has been reviewed by the Animal Care Committee of the University of Guelph for compliance with research involving animal participants (AUP #4189) and by the Research Ethics Board of the University of Guelph for compliance with federal guidelines for research involving human participants (REB # 19-06-005).

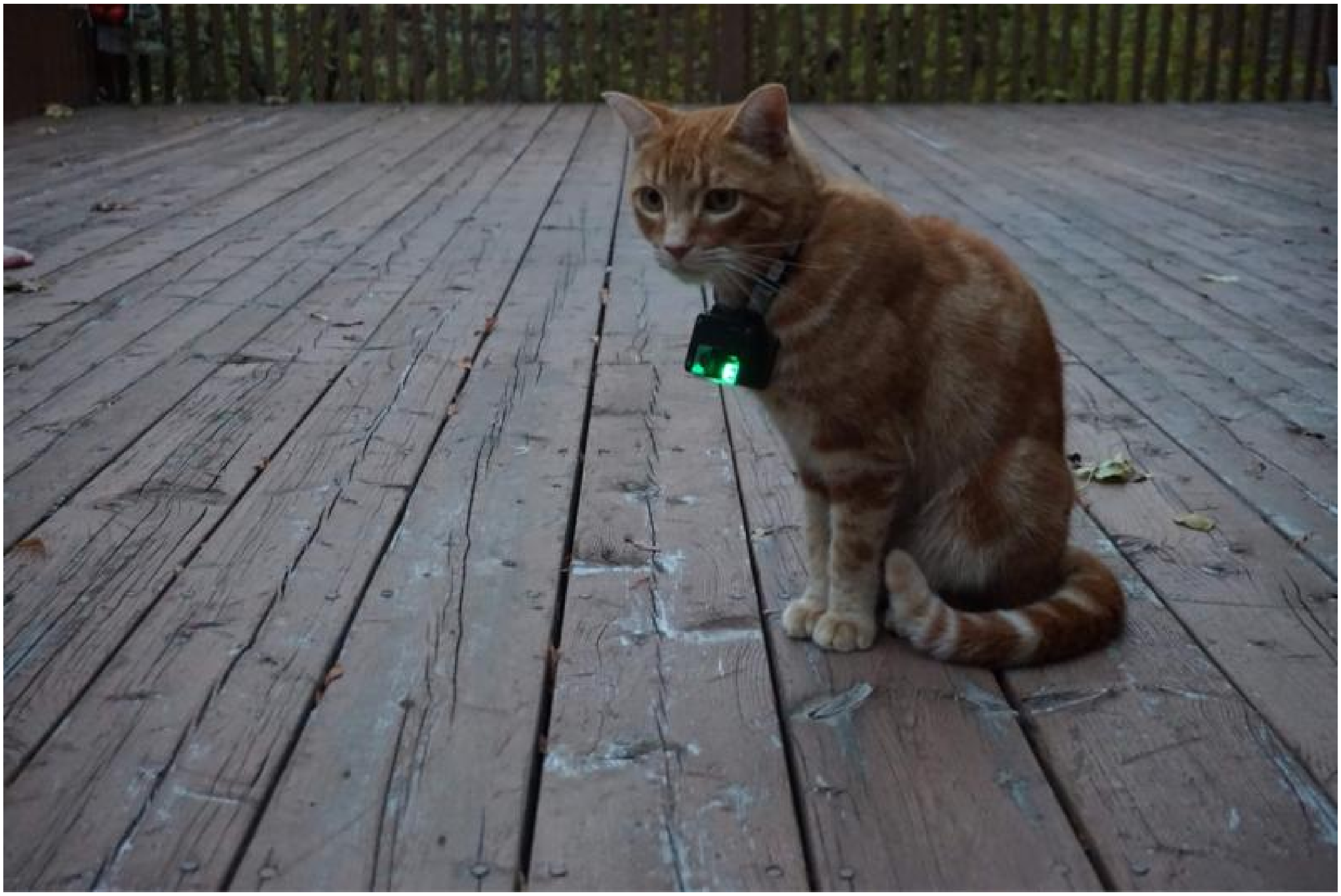

Do you live in the Kitchener-Waterloo-Cambridge-Guelph region? If YES, what is the closet major intersection to you? If NO, you do not need to go any further with the survey.

Yes

No

Do you own a cat that spends time outside unsupervised (i.e., not including in a catio, on a leash or tether, etc.)? If NO, you do not need to go any further with the survey.

Yes

No

How many cats do you own?

If you have more than one outdoor cat, please answer all of the below questions in regards to ONLY one of your outdoor cats.

Our *Catcams* weigh roughly the equivalent of an extra large egg (approximately 70 g) and can fit in the palm of your hand! The *Catcams* sit below the chin of your cat and are attached to a standard cat break-away collar (supplied by the research team). Our goal is to obtain video footage from each cat for 20 days within a 5-week period. Would you be willing to attach a *Catcam* to your cat's collar before they go outside over this time period? If NO, you don't need to go any further with the survey.

Yes

No

Does your cat regularly wear a collar?

Yes

No

Is your cat in good health? If no, please briefly explain health issues.

Yes

No

Is your cat on any medications? If so, please indicate what kind.

Yes

No

Is your cat free to go in and out of the house on their own (i.e. using a cat door)?

Yes

No

Roughly how often is your cat let outside per day?

1-2 times

2-4 times

> 4 times

When your cat is outside, roughly how much time does it spend outside in a single period? If other, please specify.

< 1 hour

1-3 hours

3-5 hours

Other

When is your cat typically outside?

Only during the day

Mostly during the day and sometimes at night

During both the day and night

Only during the night

Mostly during the night and sometimes during the day

How old is your cat?

What sex is your cat?

Male

Female

Is your cat spayed/neutered?

Yes

No

What is the approximate weight of your cat? Please specify units (lbs or kg).

Has your cat been vaccinated for Feline Leukemia Virus (FeLV)?

Yes

No

Unknown

Has your cat been vaccinated for Feline Immunodeficiency virus (FIV)?

Yes

No

Unknown

Please write your first name in the space below.

Please write your cat's name in the space below.

Please enter your phone number in the space below.

Please write your contact email in the space below.

Powered by Qualtrics
