# Supplementary material for: Home range size and habitat selection of owned outdoor domestic cats (Felis catus) in urban southwestern Ontario
Source: PeerJ. 2024 Mar 29;12:e17159. doi: 10.7717/peerj.17159 (PMC10984174; doi:10.7717/peerj.17159)
Supplement: Supplemental Information 3 [file peerj-12-17159-s003.docx]

**Supplementary Materials**

**Table of contents:**

Appendix S1: Home range with respect to cumulative GPS fixes.

Figure S.1. Relationship between the home range size and number of GPS points collected.

Appendix S2: Correlation between predictor variables.

Figure S.2. Correlation matrix.

Appendix S3 Additional details about AICc model section.

Appendix S4: Additional details about 95% KDE home range size.

Table S4. Results from the top candidate GLMs predicting what intrinsic variables influenced the 95% KDE home range size.

Table S4.1. Results of the top candidate models used to predict what extrinsic factors influenced the 95% KDE home range size.

Table S4.2. Model averaged results of what extrinsic factors influenced the 95% KDE home range size.

Appendix S5: Additional details about habitat selection.

Table S5. Model results for habitat selection within a boundary of 300 m

Appendix S6: Major roads may act as barriers for cat movement.

Figure S6. A map highlighting how roads may act as barriers for cat movement.

**Appendix S1: Home range with respect to cumulative GPS fixes**


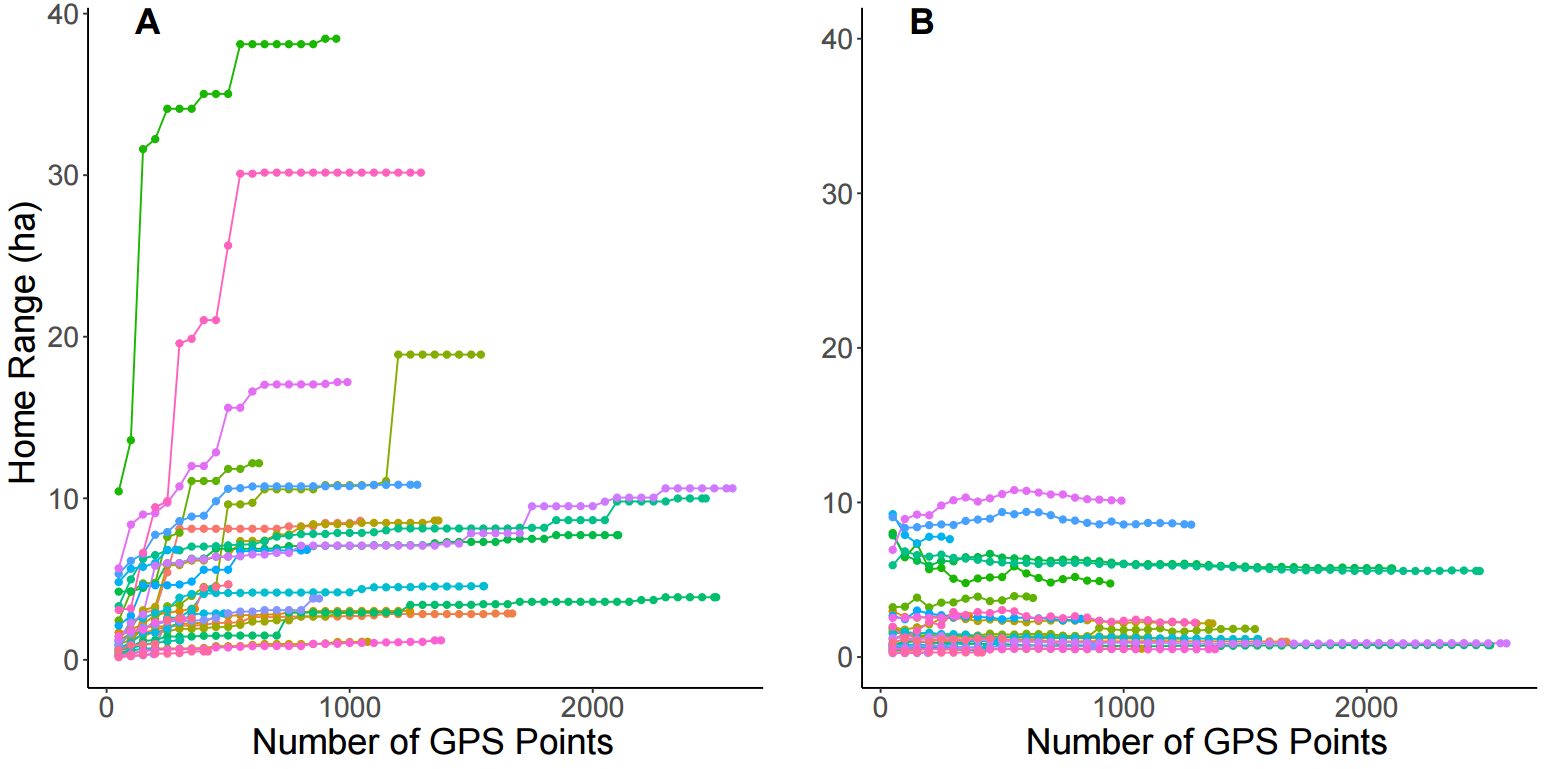


**Figure S1**. The cumulative number of GPS points taken from sampled cats (each line represents a different individual) with respect to the calculated A) 100% minimum convex polygon home range and B) 95% kernel density estimate home range.

**Appendix 2: Correlation between predictor variables**


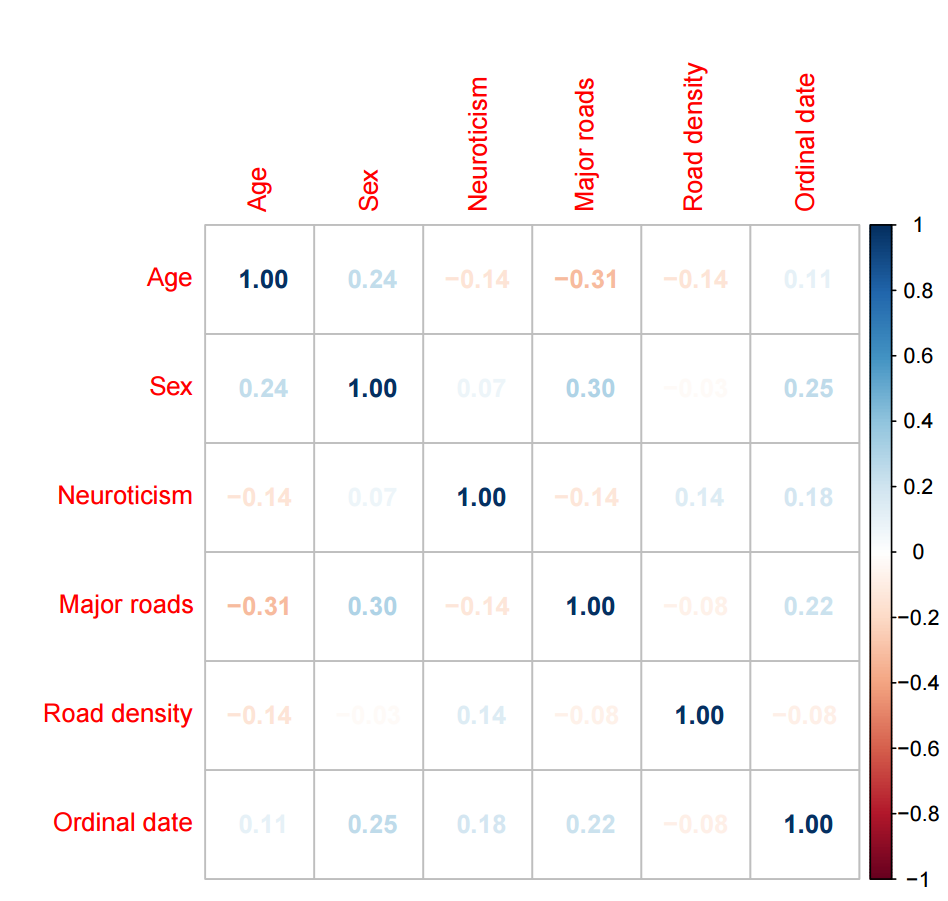


**Figure S2**. Visualization of the correlation matrix of predictor variables used to model home range size. The figure was generated using the R package “corrplot” (Wei & Simko, 2021).

**Appendix 3: Additional details about AICc model selection**

| **Table S3**. Model selection results showing all possible combinations of intrinsic factors that could influence the cat’s 100% MCP home range size. | | | | |
| --- | --- | --- | --- | --- |
| Model | df | AICc | ΔAICc | Weight |
| null | 2 | 182.7196 | 0 | 0.3625 |
| age | 3 | 184.1567 | 1.4371 | 0.1767 |
| sex | 3 | 184.7643 | 2.0447 | 0.1304 |
| neuroticism | 3 | 185.1946 | 2.4750 | 0.1052 |
| age + sex | 4 | 185.9441 | 3.2245 | 0.0723 |
| age + neuroticism | 4 | 186.7619 | 4.0423 | 0.0480 |
| neuroticism + sex | 4 | 187.3498 | 4.6302 | 0.0358 |
| neuroticism + sex + neuroticism*sex | 5 | 188.3188 | 5.5992 | 0.0221 |
| age + neuroticism + sex | 5 | 188.5881 | 5.8685 | 0.0193 |
| age + sex + age*sex | 5 | 188.8861 | 6.1665 | 0.0166 |
| age + neuroticism + sex + neuroticism*sex | 6 | 190.9256 | 8.2060 | 0.0060 |
| age + neuroticism + sex + age*sex | 6 | 191.7165 | 8.9970 | 0.0040 |
| age + neuroticism + sex + age*sex + neuroticism*sex | 7 | 194.3235 | 11.6039 | 0.0011 |

| **Table S3.1**. Model selection results showing all possible combinations of extrinsic factors that could influence the cat’s 100% MCP home range size. | | | | |
| --- | --- | --- | --- | --- |
| Model | df | AICc | ΔAICc | Weight |
| road density | 3 | 181.9789 | 0 | 0.2726 |
| null | 2 | 182.7196 | 0.7407 | 0.1882 |
| major road + road density | 4 | 182.8921 | 0.9132 | 0.1727 |
| major road | 3 | 183.4459 | 1.4670 | 0.1309 |
| time + road density | 4 | 184.3111 | 2.3322 | 0.0849 |
| time + major road + road density | 5 | 185.0394 | 3.0605 | 0.0590 |
| time | 3 | 185.1956 | 3.2167 | 0.0546 |
| time + major road | 4 | 185.9759 | 3.9970 | 0.0370 |

| **Table S3.2**. Model selection results showing all possible combinations of intrinsic factors that could influence the cat’s 95% KDE home range size. | | | | |
| --- | --- | --- | --- | --- |
| Model | df | AICc | ΔAICc | Weight |
| null | 2 | 112.9432 | 0 | 0.2831 |
| age | 3 | 113.2602 | 0.3170 | 0.2416 |
| sex | 3 | 114.9825 | 2.0393 | 0.1021 |
| neuroticism | 3 | 115.4390 | 2.4958 | 0.0813 |
| neuroticism + sex + neuroticism*sex | 5 | 115.6328 | 2.6896 | 0.0738 |
| age + neuroticism | 4 | 115.7807 | 2.8374 | 0.0685 |
| age + sex | 4 | 115.8759 | 2.9327 | 0.0653 |
| neuroticism + sex | 4 | 117.6798 | 4.7366 | 0.0265 |
| age + Neuroticism + sex + neuroticism*sex | 6 | 118.3381 | 5.3949 | 0.0191 |
| age + Neuroticism + sex | 5 | 118.6106 | 5.6673 | 0.0167 |
| age + sex + age*sex | 5 | 118.8082 | 5.8650 | 0.0151 |
| age + Neuroticism + sex + age*sex | 6 | 121.7071 | 8.7639 | 0.0036 |
| age + Neuroticism + sex + age*sex + neuroticism*sex | 7 | 121.7685 | 8.8253 | 0.0034 |

| **Table S3.3**. Model selection results showing all possible combinations of extrinsic factors that could influence the cat’s 95% KDE home range size. | | | | |
| --- | --- | --- | --- | --- |
| Model | df | AICc | ΔAICc | Weight |
| time + road density | 4 | 101.4003 | 0 | 0.3628 |
| time + major road + road density | 5 | 101.8162 | 0.41589 | 0.2947 |
| road density | 3 | 102.4178 | 1.0175 | 0.2181 |
| major road + road length | 4 | 103.6254 | 2.2251 | 0.1193 |
| time + major road | 4 | 112.1669 | 10.7666 | 0.0017 |
| time | 3 | 112.2965 | 10.8962 | 0.0016 |
| null | 2 | 112.9432 | 11.5429 | 0.0011 |
| major road | 3 | 113.6024 | 12.2021 | 0.0008 |

**Appendix 4: Additional details about home range size**

From the analysis of all 42 cats, the median 95% kernel density estimate (KDE) home range size was 1.36 ha (ranged: 0.27–11.18 ha). We did not find support for the sex hypothesis (hypothesis 1). The male and female 95% KDE home ranges showed similar variation (Brown-Forsythe test, F = 0.30, *p* = 0.59), with the median male 95% KDE home range being 2.01 ha (range: 0.27–10.16 ha) and females had a median home range size of 0.91 ha (range: 0.29–8.56 ha). We did not find evidence to support cats had a larger home range at night than during the day (hypothesis 2). During the day, the median home range size was 1.46 ha (range: 0.18–10.54 ha), while during the night the median home range size was 1.43 ha (range: 0.20–12.11 ha), which was not a significant difference (Wilcox signed rank test, *W* = 507, *p* = 0.50).

To explain what intrinsic variables could influence the size of a cat’s 95% KDE home range, there were two top models (ΔAICc < 2; Table S3.2). The top ranked model was the null model, and the second-ranked model (ΔAICc = 0.32) included age. The confidence intervals for age did overlap with zero (β= -0.08, 85% CI [-0.18, 0.14]; Table S4), suggesting little support that home range size was influenced by sex, age, and neuroticism (hypotheses 1, 3 and 5), and so we did not include these variables in the subsequent evaluation of extrinsic effects. To explain what extrinsic variables could influence the size of the home range, there were three candidate models within 2 ΔAICc of the top model (Table S4.1). Based on the model averaged coefficient estimate and confidence intervals not overlapping with zero, there was no support that traffic acted as a barrier for cats (hypothesis 4) as road density and time were both positively associated with home range size (Table S4.2).

| **Table S4.** Model results of the top candidate GLM predicting intrinsic factors that influence the 95% MCP home range size of sampled cats. Age was measured in years. | | | |
| --- | --- | --- | --- |
| Variable | Estimate | Lower 85% CI | Upper 85% CI |
| Intercept | 1.4384 | 0.7260 | 2.2114 |
| Age | -0.0824 | -0.1764 | 0.0139 |

| **Table S4.1**. Model selection results showing the top candidate models predicting what extrinsic factors influenced 95% KDE home range size of sampled cats. Road density was estimated by summing the road lengths, measured in meters, within a fixed boundary centred on each cat’s mean latitude and longitude coordinates. The predictor variable “major road” indicated the presence or absence of a major road near the cat’s home range (binary). Roads were labeled as “major” based on Google Maps’ classification, related to traffic rates, and through “ground-truthing”. Time was the ordinal date of the first GPS point recorded. | | | | |
| --- | --- | --- | --- | --- |
| Model | df | AICc | ΔAICc | Weight |
| Time + road density | 4 | 101.4003 | 0 | 0.3628 |
| Time + road density + major road | 5 | 101.8162 | 0.4159 | 0.2947 |
| Road density | 3 | 102.4178 | 1.0175 | 0.2181 |

| **Table S4.2**. Model-averaged results candidate GLMs predicting factors that influenced 95% KDE home range size of sampled owned cats. Road density was estimated by summing the road lengths, measured in meters, within a fixed boundary centred on each cat’s mean latitude and longitude coordinates. The variable “major road” indicated the presence or absence of a major road near the cat’s home range (binary). Roads were labeled as “major” based on Google Maps’ classification, related to traffic rates, and through “ground-truthing”. | | | |
| --- | --- | --- | --- |
| Variable | Estimate | Lower 85% CI | Upper 85% CI |
| Time | 0.0063 | 0.0017 | 0.0111 |
| Road density | 0.0073 | 0.0046 | 0.0103 |
| Major road | -0.9524 | -1.6809 | -0.0595 |

**Appendix 5: Additional details about habitat selection**

Based on the random points generated within a 300m buffer around the houses of 41 cats, the most abundant habitat types available were impervious surfaces (60%), then roads (24%), greenspaces (11%), and the least abundant habitat type was agricultural land (5%). Within the buffers, greenspaces were comprised of recreational parks (58%), and various natural habitats (treed swamps [14%], thicket swamps [13%], deciduous forests [9%], marshlands [5%], hedge rows [0.9%], forests [0.3%], and coniferous forests [0.2%]). Results from the GLMM provided some support for our hypotheses that traffic and coyotes could act as barriers for cat movement as cats selected for impervious surfaces (73% of used locations), showed a weak avoidance for roads (22% of used locations), a strong an avoidance of greenspaces (4% of used locations), and a stronger avoidance for agricultural land (1% of used locations) in proportion to their availability (Table S5, Figure 6).

**Table S5**. The regression coefficient (β) from a binomial logistic regression model for cat habitat selection within a boundary of 300 m centered on their owner’s house, with impervious surfaces as a reference level. Habitat types were obtained from the Southern Ontario Land Resource Information System (SOLRIS) v.3 (Ontario Ministry of Natural Resources and Forestry, 2019).

| Habitat Type | Coefficient | Lower 95% CI | Upper 95% CI | *p* |
| --- | --- | --- | --- | --- |
| Impervious | 0.64 | 0.62 | 0.67 | < 0.0001 |
| Greenspaces | -1.27 | -1.33 | -1.22 | < 0.0001 |
| Roads | -0.15 | -0.17 | -0.12 | < 0.0001 |
| Agricultural | -2.17 | -2.28 | -2.06 | < 0.0001 |

**Appendix 6: Major roads may act as barriers for cat movement**


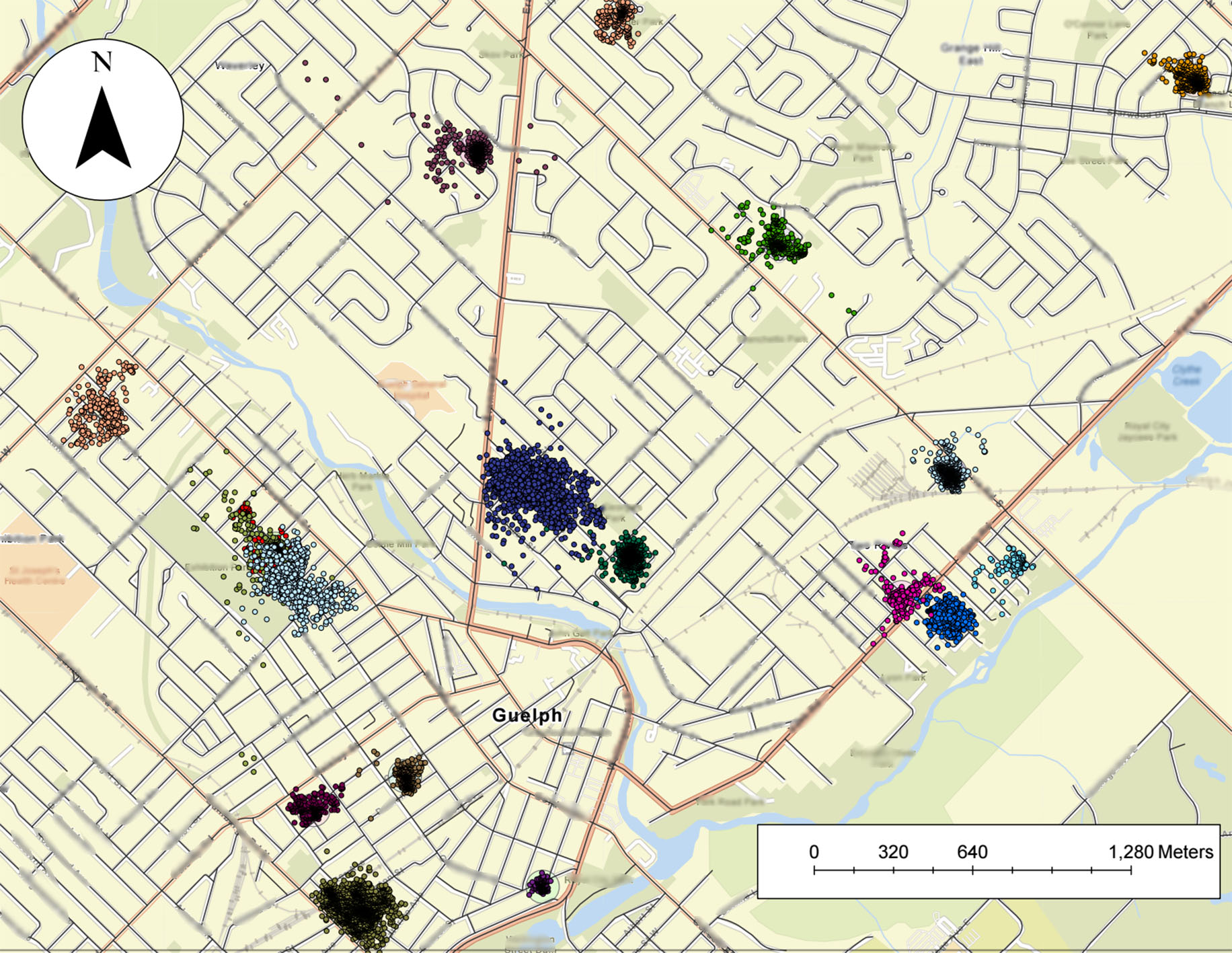


**Figure S6**. Map of GPS points from owned cats living in the Guelph region. This map also shows minor (black) and major (orange) roads in the city, the latter of which appeared to act as a barrier for movement.
