# Supplementary material for: Home range size and habitat selection of owned outdoor domestic cats (Felis catus) in urban southwestern Ontario
Source: PeerJ. 2024 Mar 29;12:e17159. doi: 10.7717/peerj.17159 (PMC10984174; doi:10.7717/peerj.17159)
Supplement: Supplemental Information 4 [file peerj-12-17159-s004.pdf]

Below each cat behaviour or personality characteristic there is a ranking scale ranging from "strongly disagree" **to** "strongly agree". Please rate your cat on whether they demonstrate each behaviour or personality characteristic. Since each personality characteristic might differ from your own personal definition of the word, please use the definition we have provided here.

**NOTE:** Please answer ALL of the questions, as failing to do so will result in an inability to asses your cat's personality.

This project has been reviewed by the Animal Care Committee of the University of Guelph for compliance with research involving animal participants (AUP #4189) and by the Research Ethics Board of the University of Guelph for compliance with federal guidelines for research involving human participants (REB # 19-06-005).

What is your cat's name?

What is the **ID code** given to you by the research team?

Please rate your cat on whether they demonstrate each behaviour or personality characteristic.

| | Strongly Disagree | Disagree | Somewhat Disagree | Neutral | Somewhat Agree | Agree | Strongly Agree |
|---|---|---|---|---|---|---|---|
| Vigilant – Watchful, observant; spends a lot of time attending to his/her surroundings. | ○ | ○ | ○ | ○ | ○ | ○ | ○ |
| Stable – Calmly reacts to his/her environment. | ○ | ○ | ○ | ○ | ○ | ○ | ○ |
| Bold – daring, not restrained or tentative, doesn't hesitate. | ○ | ○ | ○ | ○ | ○ | ○ | ○ |
| Clumsy – relatively awkward or uncoordinated during movements, e.g. when walking, climbing, or playing. | ○ | ○ | ○ | ○ | ○ | ○ | ○ |
| Defiant – assertive, difficult or challenges the usual dominance order with other cats or people in your household. continues these actions despite unfavourable | ○ | ○ | ○ | ○ | ○ | ○ | ○ |
| Gentle – responds to others (people, cats or other animals) in an easy going manner, which is not rough or threatening. | ○ | ○ | ○ | ○ | ○ | ○ | ○ |
| Constrained – controlled and not very impulsive. | ○ | ○ | ○ | ○ | ○ | ○ | ○ |
| Inquisitive – is drawn to new situation, objects, or animals and behaves as if s/he wishes to learn more about others or objects within view. | ○ | ○ | ○ | ○ | ○ | ○ | ○ |
| Inventive – is more likely than other cats to do new things | ○ | ○ | ○ | ○ | ○ | ○ | ○ |

| | Strongly Disagree | Disagree | Somewhat Disagree | Neutral | Somewhat Agree | Agree | Strongly Agree |
|---|---|---|---|---|---|---|---|
| including novel or problem solving behaviours (e.g. opening a cupboard or entering bags or boxes) | | | | | | | |
| Irritable – often seems in a bad mood or is impatient and easily provoked to anger or other agnostic or threatening/aggressive behaviour. | ○ | ○ | ○ | ○ | ○ | ○ | ○ |
| Distractible – easily distracted and has a short attention span. | ○ | ○ | ○ | ○ | ○ | ○ | ○ |
| Erratic – Inconsistent or widely varying in behaviour and moods. | ○ | ○ | ○ | ○ | ○ | ○ | ○ |
| Solitary – spends times alone or avoids company by choice. | ○ | ○ | ○ | ○ | ○ | ○ | ○ |
| Impulsive – displays spontaneous or sudden behaviour that was not anticipated. | ○ | ○ | ○ | ○ | ○ | ○ | ○ |
| Independent – behaviour not influenced or controlled by other animals, events or things. | ○ | ○ | ○ | ○ | ○ | ○ | ○ |
| Jealous – often troubled by other cats, pets or people who are in a desirable or advantageous situation such as having food, a choice location, or access to social situation. May attempt to disrupt activities of advantageous cats. | ○ | ○ | ○ | ○ | ○ | ○ | ○ |
| Fearful of other cats – Retreats readily or | ○ | ○ | ○ | ○ | ○ | ○ | ○ |

| | Strongly Disagree | Disagree | Somewhat Disagree | Neutral | Somewhat Agree | Agree | Strongly Agree |
|---|---|---|---|---|---|---|---|
| moves away from other cats. | | | | | | | |
| Persevering – Continues in a course of action, task, or strategy for a long time or continues despite opposition. | ○ | ○ | ○ | ○ | ○ | ○ | ○ |
| Greedy – Excessively desirous or covetous of food, favoured locations, or other resources and unwilling to share these resources with other cats/pets. | ○ | ○ | ○ | ○ | ○ | ○ | ○ |
| Friendly to other cats – Initiates proximity with or getting close to other cats; approached other cats readily and in a friendly manner, (e.g. with purrs, rubs). | ○ | ○ | ○ | ○ | ○ | ○ | ○ |
| Submissive – Often gives in or yields to another cat/pet. | ○ | ○ | ○ | ○ | ○ | ○ | ○ |
| Dominant – Controlling, exerting forcefulness, powerful with respect to other. | ○ | ○ | ○ | ○ | ○ | ○ | ○ |
| Predictable – Consistent and steady behaviour over extended periods of time; sticks to a behavioural routine or set of activities; Does little that is unexpected or deviated from its usual behaviour. | ○ | ○ | ○ | ○ | ○ | ○ | ○ |
| Suspicious – Not trusting; does not approach easily, e.g. human or animal visitors in the house) | ○ | ○ | ○ | ○ | ○ | ○ | ○ |
| Affectionate – Warm attachment to or | ○ | ○ | ○ | ○ | ○ | ○ | ○ |

| | Strongly Disagree | Disagree | Somewhat Disagree | Neutral | Somewhat Agree | Agree | Strongly Agree |
|---|---|---|---|---|---|---|---|
| closeness with other cats, pets or people e.g. grooming, touching, or lying next to other cats, pets or people). | | | | | | | |
| Insecure – Seems scared easily, jumpy and fearful in general). | ○ | ○ | ○ | ○ | ○ | ○ | ○ |
| Bullying – Overbearing and intimidating towards other cats. | ○ | ○ | ○ | ○ | ○ | ○ | ○ |
| Curious – Seeks out or investigates novel situations. | ○ | ○ | ○ | ○ | ○ | ○ | ○ |
| Aimless – Seems to behave without any clear purpose or direction. | ○ | ○ | ○ | ○ | ○ | ○ | ○ |
| Tense – Shows restraint in movement and posture, e.g. almost frozen in position. | ○ | ○ | ○ | ○ | ○ | ○ | ○ |
| Fearful of people – Retreats readily or moves away from people, especially new people entering the house. | ○ | ○ | ○ | ○ | ○ | ○ | ○ |
| Cool – Unaffected by emotions and usually undisturbed, assured, and calm. | ○ | ○ | ○ | ○ | ○ | ○ | ○ |
| Aggressive to people – (Reacts in a hostile way or attempts to attack/threaten people). | ○ | ○ | ○ | ○ | ○ | ○ | ○ |
| Calm – (Not easily distributed by changes in the environment). | ○ | ○ | ○ | ○ | ○ | ○ | ○ |
| Excitable – Overreacts to changes in environment. | ○ | ○ | ○ | ○ | ○ | ○ | ○ |

| | Strongly Disagree | Disagree | Somewhat Disagree | Neutral | Somewhat Agree | Agree | Strongly Agree |
|---|---|---|---|---|---|---|---|
| Friendly to people – Initiates proximity or closeness to people by approaching readily and in a friendly manner, e.g. purring and/or rubbing against legs; approaches new people coming into the house. | ○ | ○ | ○ | ○ | ○ | ○ | ○ |
| Playful – Initiates and engages in non-aggressive play behaviour with objects, which may seam meaningless. | ○ | ○ | ○ | ○ | ○ | ○ | ○ |
| Vocal – Frequently and readily vocalizes. | ○ | ○ | ○ | ○ | ○ | ○ | ○ |
| Decisive – Seems determined and purposeful in his/her activities. | ○ | ○ | ○ | ○ | ○ | ○ | ○ |
| Self-assured – Moves in seemingly confident, well-coordinated, and relaxed manner. | ○ | ○ | ○ | ○ | ○ | ○ | ○ |
| Anxious – Interested but fearful and uneasy; seems to change his/her mind about approach or withdrawal. | ○ | ○ | ○ | ○ | ○ | ○ | ○ |
| Trusting – Not suspicious and approaches easily, e.g. human or animal visitors in the house. | ○ | ○ | ○ | ○ | ○ | ○ | ○ |
| Active – Moves frequently, e.g. often walks, runs, stalks. | ○ | ○ | ○ | ○ | ○ | ○ | ○ |
| Cooperative – Is compliant; willingly behaves when asked to do something. | ○ | ○ | ○ | ○ | ○ | ○ | ○ |
| Shy – Reluctant to approach other | ○ | ○ | ○ | ○ | ○ | ○ | ○ |

| | Strongly Disagree | Disagree | Somewhat Disagree | Neutral | Somewhat Agree | Agree | Strongly Agree |
|---|---|---|---|---|---|---|---|
| animals or people, novel objects, or new | | | | | | | |

Powered by Qualtrics
